# Expression analysis in a rat psychosis model identifies novel candidate genes validated in a large case–control sample of schizophrenia

**DOI:** 10.1038/tp.2015.151

**Published:** 2015-10-13

**Authors:** A Ingason, I Giegling, A M Hartmann, J Genius, B Konte, M Friedl, Schizophrenia Working Group of the Psychiatric Genomics Consortium (PGC), S Ripke, P F Sullivan, D St. Clair, D A Collier, M C O'Donovan, K Mirnics, D Rujescu

**Affiliations:** 1 Department of Psychiatry, University of Halle-Wittenberg, Halle, Germany; 2 Department of Psychiatry, Ludwig-Maximilians-University, Munich, Germany; 3 Analytical and Translational Genetics Unit, Department of Medicine, Massachusetts General Hospital, Boston, MA, USA; 4 Department of Genetics, University of North Carolina at Chapel Hill, Chapel Hill, NC, USA; 5 Department of Mental Health, University of Aberdeen, Aberdeen, UK; 6 King's College London, Social, Genetic and Developmental Psychiatry Centre, Institute of Psychiatry, London, UK; 7 Institute of Psychological Medicine and Clinical Neurosciences, MRC Centre for Neuropsychiatric Genetics and Genomics, Cardiff University, Cardiff, UK; 8 Department of Psychiatry, Kennedy Center for Research on Human Development, Vanderbilt University, Nashville, TN, USA

## Abstract

Antagonists of the N-methyl-D-aspartate (NMDA)-type glutamate receptor induce psychosis in healthy individuals and exacerbate schizophrenia symptoms in patients. In this study we have produced an animal model of NMDA receptor hypofunction by chronically treating rats with low doses of the NMDA receptor antagonist MK-801. Subsequently, we performed an expression study and identified 20 genes showing altered expression in the brain of these rats compared with untreated animals. We then explored whether the human orthologs of these genes are associated with schizophrenia in the largest schizophrenia genome-wide association study published to date, and found evidence for association for 4 out of the 20 genes: *SF3B1*, *FOXP1*, *DLG2* and *VGLL4*. Interestingly, three of these genes, *FOXP1*, *SF3B1* and *DLG2*, have previously been implicated in neurodevelopmental disorders.

## Introduction

Schizophrenia is a debilitating major psychiatric disorder^1^ associated with severely reduced quality of life and reduced life expectancy among those affected.^2, 3^ The disorder is characterized by various behavioral symptoms both positive (including hallucinations, formal thought disorder and paranoia) and negative (incl. apathy, social withdrawal and reduced mood), as well as cognitive symptoms (incl. impaired memory and attention span).^1^ A major factor contributing to the reduced quality of life and reduced life expectancy in schizophrenia is its resistance to treatment. Although antipsychotic medications can alleviate some of the symptoms in patients, notably the positive symptoms, there is no cure available for schizophrenia, and most patients who respond to current treatments fail to cope fully in society, as the negative and cognitive symptoms are poorly treated. Whereas conventional antipsychotics act by blocking dopamine receptors, the last two decades have seen growing evidence linking defects in glutamatergic neurotransmission to schizophrenia pathology.^4, 5^ The primary evidence in this regard is the observation that noncompetitive antagonists of the N-methyl-D-aspartate (NMDA)-type glutamate receptor, including ketamine and phencyclidine, exacerbate manifestations of schizophrenia in patients and induce positive and negative symptoms as well as cognitive impairments in healthy individuals.^6, 7, 8^ Rodent animal models using MK801 as an antagonist show supporting evidence to the NMDA receptor hypofunction model in schizophrenia on a molecular, cellular, functional and behavioral level including altered expression of NMDA receptor subunits, decrease in the relative number of a specific subset of GABAergic interneurons, evidence of altered recurrent inhibition of pyramidal cells and cognitive deficits associated with hippocampal dysfunction, resembling findings in patients.^9^

Grunze *et al.*^10^ found that GABAergic interneurons in the hippocampus are especially sensitive to NMDA receptor antagonists and postulated that reduction in GABAergic inhibition could mediate the cognitive impairment seen in schizophrenia or induced in healthy subjects by ketamine and other partial NMDA antagonists, respectively. This hypothesis found some support in two functional brain-imaging studies showing reduced hippocampal activation in schizophrenia subjects while performing a memory task because of a high hippocampal baseline activity.^11, 12^ Harris *et al.*^13^ found that adult mice that had been treated neonatally with MK-801, an NMDA receptor antagonist, had reduced volume and neuronal number within the hippocampus, altered hippocampal NMDA receptor (NR1 subunit) expression and showed prepulse inhibition deficits and increased locomotor activity, reminiscent of alterations reported in schizophrenia. Frolich and Van Horn^14^ provide a recent review of the utility of the ketamine model of schizophrenia and the critical role of GABAergic interneurons.

The etiology of schizophrenia involves a strong genetic component with heritability estimates ranging from 60 to 80%.^15, 16^ Prominent candidate genes, identified by linkage and fine-mapping studies in the early 2000s and believed to be involved in schizophrenia pathology, include several members of the glutamatergic signaling network, such as Neuregulin 1 (ref. 17) and its receptor ErbB4 (ref. 18) that regulate the activity of NMDA receptors at postsynaptic sites.^19, 20^ The introduction of single-nucleotide polymorphism microarrays has pushed forward the field of genetics in the last years, as genotypes from hundreds of thousands of markers across the genome can be obtained from a single assay. This has led to the identification of several novel genes involved in schizophrenia etiology, including *ZNF804A,*^21, 22^ *NRGN,*^23^ *TCF4,*^23^ *VRK2* (ref. 24) several genes in the major histocompatibility complex region on 6p,^23, 25, 26^ and, most recently, by combining results from 52 case–control studies in a meta-analysis including over 30 000 affected and even more controls, over 100 novel schizophrenia-associated loci were identified.^27^ These microarrays have also allowed the discovery of genomic copy number variations (CNVs) that associate with schizophrenia, implicating further genes such as *NRXN1,*^28^ *CHRNA7* (refs. 29, 30) and *CYFIP1* (ref. 29) in the pathology of the disorder.

Here, we have performed an expression study on rats chronically treated with low doses of the NMDA receptor antagonist MK-801, and identified a set of 20 genes differentially expressed in the hippocampus of these rats compared with controls. Hypothesizing that the orthologs of these genes are involved in schizophrenia pathophysiology in humans, we then investigated the association of markers mapping to the human orthologs of these genes in the genome-wide association study (GWAS) results for schizophrenia from the large Psychiatric Genetics Consortium (PGC) meta-analysis.^27^ In addition, we searched a gene-wide typed schizophrenia case–control sample for CNVs overlapping exons at these 20 loci.

## Materials and methods

### Animal samples

Male Long Evans rats (*n*=24; age 35±1 days; initial weight 121–148 g) were housed in groups of four in cages in a temperature-controlled room (23 °C), with a 12/12-h light/dark cycle and with food and water provided *ad libitum*. They received daily intraperitoneal injections (10 ml kg^−1^ body weight, 0.9% saline as vehicle) of either 0.02 mg kg^−1^ body weight (+)-MK-801 (*n*=12) or 0.9% saline (placebo, *n*=12) for 20 days during the light phase. While under deep CO_2_ anesthesia, rats were killed by decapitation 24 h following the last drug administration. Further details are provided in Rujescu *et al.*^31^ All manipulations were performed in strict accordance with the current versions of the US and German Law for the Protection of Animals (approval ID: 209.1/211-2531-78/03 Regierung von Oberbayern, Munich, Germany).

#### Expression analysis

Hippocampi were isolated from brains of MK-801-treated animals (*n*=12) and controls (*n*=12), shock-frozen in liquid nitrogen and immediately stored at –80 °C until further processing. Tissue (30–80 mg) was homogenized in QIAzol Lysis Reagent (Qiagen, Hilden, Germany) using ultra thurrax. Total RNA was extracted with the RNeasy Lipid Tissue Mini Kit (Qiagen) according to the manufacturer's instructions and stored at –80 °C. RNA concentration and quality was estimated using a UV spectrometer and an ethidium bromide-stained agarose gel. Equal amounts of RNA of three animals conferring to one experimental group were pooled, and 4.07±0.7 μg RNA of each of the resulting eight pools (four pools of three MK-801-treated animals and four pools of three control animals) hybridized to one array (GeneChip Rat Genome 230 2.0 Array, Affymetrix, Santa Clara, CA, USA). Expression data were normalized using the robust multiarray average method in GenePattern,^32^ and *P*-values were established using a two-tailed *t*-test.

### Human samples

#### PGC sample

The PGC schizophrenia GWAS sample includes 34 241 schizophrenia cases and 45 604 control individuals from 49 different sites, and 1235 parent affected-offspring trios. Details of the sample and the PGC schizophrenia GWAS are provided in Ripke *et al.*^27^

#### Munich sample

*Schizophrenia patients:* In total, 625 (394 men and 231 women, aged 18–70) individuals with schizophrenia were ascertained from the Munich area in Germany. All were of German (both parents German) or Central European (either or both parents non-German Central European) descent, and had a diagnosis of schizophrenia according to both the Diagnostic and Statistical Manual of Mental Disorders, 4th Edition (DSM-IV) and International Statistical Classification of Diseases and Related Health Problems, 10th revision (ICD-10). Detailed medical and psychiatric histories were collected, including the Structured Clinical Interview for DSM-IV (SCID), to evaluate lifetime Axis I and II diagnoses.^33, 34^ Four physicians and one psychologist rated the SCID interviews and all measurements were double-rated by a senior researcher. Exclusion criteria included a history of head injury or neurological diseases. All case participants were outpatients or stable in-patients.

*Healthy controls:* In total, 539 (246 men and 293 women, aged 19–72) unrelated volunteers of German descent (that is, both parents German) were randomly selected from the general population of Munich, Germany, and contacted by mail. To exclude subjects with central neurological diseases and psychotic disorders or subjects who had first-degree relatives with psychotic disorders, several screenings were conducted before the volunteers were enrolled in the study. First, subjects who responded were screened by phone for the absence of neuropsychiatric disorders. Second, detailed medical and psychiatric histories were assessed for both themselves and their first-degree relatives by using a semistructured interview. Third, if no exclusion criteria were fulfilled, they were invited to a comprehensive interview including the Structured Clinical Interview for DSM-IV (SCID I and SCID II)^33, 34^ to validate the absence of any lifetime psychotic disorder. In addition, the Family History Assessment Module^35^ was conducted to exclude psychotic disorders among first-degree relatives. Furthermore, a neurological examination was conducted to exclude subjects with current central nervous system impairment. In the case that the volunteers were older than 60 years, the Mini Mental Status Test^36^ was performed to exclude subjects with possible cognitive impairment.

Written informed consent was obtained from all subjects after a detailed and extensive description of the study, which was approved by the local ethics committee and carried out in accordance to the ethical standards laid down in the Declarations of Helsinki.

*Genotyping:* The Munich samples were genotyped on the Illumina HumanHap300 arrays. Blood was drawn and DNA isolated following standard procedures as described in Stefansson *et al.*^29^ and Need *et al.*^37^ The majority of samples (*n*=790; 437 cases and 353 controls) were genotyped as part of a schizophrenia case–control GWAS described in detail in Need *et al.*^37^ Another 374 samples (188 cases and 186 controls) were genotyped as part of the SGENE study, a multicenter collaboration to find schizophrenia susceptibility genes; the genotyping of these samples is described in detail in Stefansson *et al.*^29^ We used the PennCNV algorithm^38^ to derive CNV calls from the Munich sample. The list of CNV calls was then refined by removing calls from sample outliers as well as calls spanning fewer than 10 consecutive markers, and by joining calls occurring in the same sample close-by on the same chromosome if the total length of the interval(s) between the calls was less than 50% of their combined length including the interval(s).

## Results

**Figure 1:**
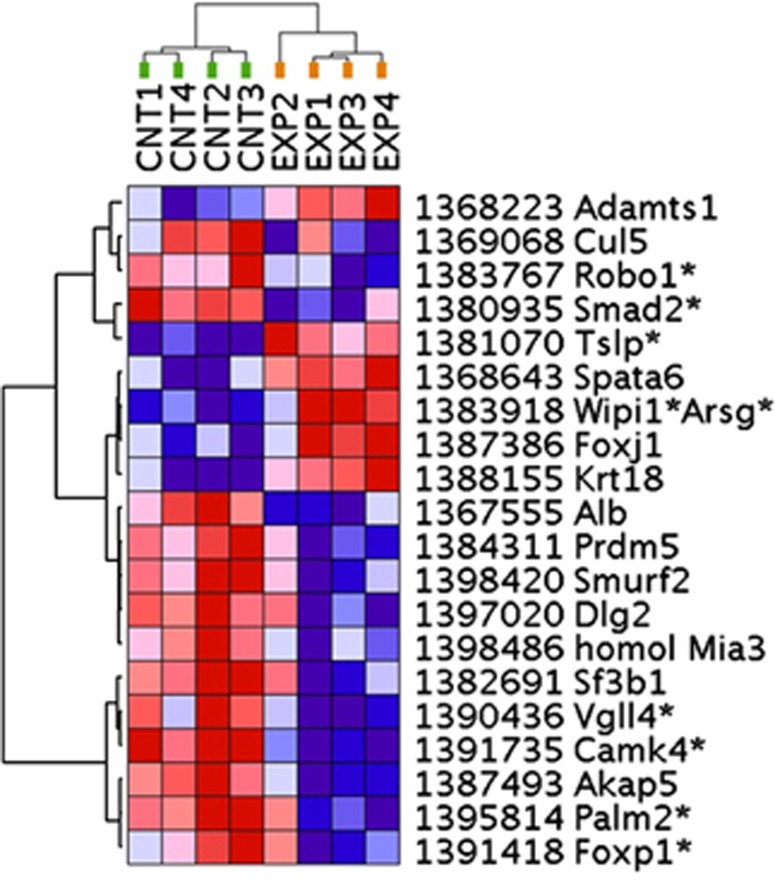
Differentially expressed transcripts in the MK801 treatment. Transcripts were subjected to a two-way (horizontal: genes; vertical: samples) unsupervised clustering using GenePattern and Euclidian distance calculations. Individual genes are denoted by microarray probe numbers and gene symbols, *indicates when a transcript is in close physical proximity of a gene. Gene expression differences are color-coded (red—increased expression; blue—decreased expression), and the intensity of the color corresponds to the magnitude of gene expression change. Note that this panel of genes separates the experimental and control samples into two distinct clusters.

To define a list of truly differentially expressed genes between the MK801-treated and control animals, we first performed an experimental analysis at various levels of stringency based on |ALR| (absolute average log2 ratio; magnitude of change) and *P* (probability of random occurrence). For each level of stringency we derived the number of transcripts differentially expressed in the real experiment (e1–e2–e3–e4 versus c1–c2–c3–c4, where e1–4 and c1–4 denote the four pools of each three MK801-treated animals and four pools of three control animals, respectively) and the number of transcripts differentially expressed in three permutation comparisons (Boot 1, c1–c2–e3–e4 versus c3–c4–e1–e2; Boot 2, c1–c3–e2–e4 versus c2–c4–e1–e3; Boot 3, c1–c4–e2–e3 versus c2–c3–e1–e4). The logic here is that if the manipulation is the biggest contributor to the effect, the effect will be canceled out in the permutation comparisons, whereas if other factors are responsible for most of the effect the permutation comparisons will produce equally many differentially expressed transcripts as the real experiment. We used the results to calculate a false discovery rate (FDR), and accordingly chose to use the stringency where the FDR is lowest; the results are summarized in Table 1. Using |ALR|>0.585 (corresponding to 50% change) and *P*<0.05 gave the lowest FDR, at 34.5%, from 74 differentially expressed transcripts (Table 1 and Figure 1).

To further refine this list, we compared all four MK801-treated animals individually against all four control animals (in total 16 comparisons), and considered only those transcripts reaching 50% change (|ALR|=0.585) in at least nine comparisons as truly differentially expressed; this yielded 20 transcripts (see Table 2). Some of these transcripts are from unknown genes; in these cases we looked up the sequences and blatted against the rat genome on the UCSC Genome Browser (RGSC 6.0/rn6). In all instances these sequences mapped immediately downstream of a known rat gene (see Table 2).

Next, we looked up the corresponding human orthologs in the PGC samples to see single-nucleotide polymorphism association results from the PGC schizophrenia GWAS^27^ (see Table 3). We defined start and stop for the single-nucleotide polymorphism lookup region for each gene 20 kb from the end of the RefSeq sequence reaching furthest in each direction. Using the total number of PGC single-nucleotide polymorphisms within each lookup region, we calculated gene-wide significance as the *P*-value for the most significantly associated marker multiplied with number of markers in the gene lookup region. We found markers reaching gene-wide significance at 4 out of the 20 lookup regions: *SF3B1*, *FOXP1*, *DLG2* and *VGLL4*. For three of these genes (*SF3B1*, *DLG2* and *FOXP1*) we observe single marker associations reaching significance levels close to or even surpassing the threshold for genome-wide significance (Table 3).

We also looked in a genome-wide typed schizophrenia case–control sample for deletions and duplications affecting any of the 20 human genes and found one duplication in *ROBO1* in a schizophrenia subject (chr3:79098154-79280400), and two deletions at *DLG2*, one in a schizophrenia subject and the other in a control individual (chr11:82698224-82899038 and chr11:83450214-83671604, respectively).

## Discussion

Although GWASs provide a powerful hypothesis-free strategy for identifying genetic variations underlying disease and other traits, they require associations to reach above the high threshold of genome-wide significance (usually *P*=1 × 10^−7^–1 × 10^−8^) to be regarded as true because of the large number of markers tested. This requirement likely excludes many real association signals—along with statistical noise—some of which could be identified by applying a hypothesis-driven filter that would preclude many irrelevant genes/markers from the GWAS and thus lower the required significance threshold.

The use of NMDA receptor antagonists such as phencyclidine, ketamine and MK801 is a well-established method to model a psychosis-like state in rodents.^31, 39^ The rat model used in this study was shown previously to express psychosis-related symptoms on molecular, physiological, cellular and behavioral levels.^31^ In the current study, we looked at differences in expression on a whole-transcriptome level in the hippocampus of this animal model. Our working hypothesis is that psychosis-like symptoms induced by chronic partial blocking of NMDA receptors are at least in part mediated by altered gene expression in brain regions such as the hippocampus—investigated here—and, consequently, that genetic variation affecting the expression of human orthologs of these genes can influence risk of developing schizophrenia in humans. To test this hypothesis, we have identified the most markedly differentially expressed transcripts between the hippocampi of the MK801-treated animals and control animals, and subsequently looked at evidence from the largest meta-analysis of schizophrenia GWAS published to date to see whether markers in these genes associate with schizophrenia in humans. We also looked for CNVs at these gene loci in a case–control sample, as deletions, duplications and disruptions (partial exonic deletions or duplications) of genes have in many cases been shown to alter their expressions, usually in a dosage-dependant manner.

Following several steps aimed at identifying truly differentially expressed genes between the hippocampi of MK801-treated and control animals, we ended up with a list of 20 transcripts from the array, corresponding to 20 rat genes, as shown in Table 2. When looking at the PGC GWAS results for the human orthologs of these genes, we found that four of them included markers showing gene-wide significant associations with schizophrenia (Table 3). As regards rare CNVs in our own schizophrenia case–control sample, we found two partial deletions of the DLG2 locus (one case and one control) and one case duplication affecting the ROBO1 locus; all three CNVs were exon-disrupting.

### Candidate genes found by cross-checking expression results from the animal model with schizophrenia GWAS

*SF3B1* encodes splicing factor 3b subunit 1, a component of the RNA splicing machinery, and mutations in the gene have been found to underlie myelodysplastic syndromes that predispose to leukemia.^40^ Besides this, Sf3b1 was shown to be prominently involved in processes for adult neurogenesis. Neural stem cells and neurogenesis persist in the adult mammalian brain subventricular zone. Cells born in the rodent subventricular zone migrate to the olfactory bulb where they differentiate into interneurons. Lim *et al.*^41^ expanded the catalog of cell cycle components, transcription factors including *Sf3b1* and migration genes for adult subventricular zone neurogenesis, and revealed RNA splicing and chromatin remodeling as prominent biological processes for these germinal cells.^41^ Furthermore, SF3B1 is associated with PQBP1 (Polyglutamine-binding protein 1), which is a highly conserved protein associated with neurodegenerative disorders and is linked to intellectual disability. Wang *et al.*^42^ showed that loss of functional PQBP1 reduced SF3B1 substrate mRNA association and led to significant changes in alternative splicing patterns. Depletion of PQBP1 in primary mouse neurons reduced dendritic outgrowth and altered alternative splicing of mRNAs enriched for functions in neuron projection development. Disease-linked PQBP1 mutants were deficient in splicing factor associations and could not complement neurite outgrowth defects.^42^

Interestingly, *SF3B1* is one of the 108 genome-wide significant loci identified in the latest schizophrenia meta-analysis published by PGC.^27^ In the PGC meta-analysis, this locus stretches over roughly 300-kb region (Chr2:198.2–198.5 Mb in human genome reference build 37), in which many markers reach beyond the *P*=5 × 10^−8^ threshold for genome-wide significance, although rs6434928 shows the strongest association. Whereas this region includes several genes, our results point to *SF3B1* as the likely candidate through which the genetic risk for schizophrenia is conveyed.

*DLG2* is a member of a family of membrane-associated guanylate kinases, which function as scaffolding proteins at the postsynaptic density and are involved in NMDA receptor signaling. Recently, Kirov *et al.*^43^ found *de novo* CNVs disrupting *DLG2* (and other postsynaptic genes) in schizophrenia probands, and, in a follow-up meta-analysis including published CNV studies, found suggestive evidence for association of CNVs affecting *DLG2* with schizophrenia.^43^ In a more recent study on *de novo* SNV mutations in schizophrenia, the same group of researchers found a *de novo* splice site mutation in *DLG2* in an affected proband.^44^ Furthermore, another *DLG* family gene, *DLG1*, is contained within a recurrent deletion at 3q29, which is also associated with schizophrenia.^45, 46^ We found two exon-disrupting CNVs affecting DLG2, but only one of them was in a case subject; therefore, in this respect our findings are not supportive of association (although this could be a false-negative finding as the sample studied by Kirov *et al.*^43^ was much larger).

*FOXP1* encodes a forkhead transcription factor that has been postulated to have an important role in language development as well as its better known family member *FOXP2*. Recently, *de novo* mutations and deletions affecting *FOXP1* were reported in subjects with intellectual disability, autism and language impairment.^47, 48^

*VGLL4* encodes vestigial-like 4, a protein that regulates gene expression in myocytes^49^ and acts as a tumor suppressor in gastric cancer through counteracting oncogenes.^50^ Its function in the brain has not been described.

Within the *ROBO1* locus the strongest association with schizophrenia in the PGC sample was *P*=7.4 × 10^−5^ for rs188858898; however, as there were over 6000 markers typed (or imputed) in this locus, the association failed to reach gene-wide significance. We, however, also found a duplication disrupting *ROBO1* in a schizophrenia-affected subject from our own case–control sample. Interestingly, the International Schizophrenia Consortium reported two duplications at the ROBO1 locus in their 2008 study involving several thousand schizophrenia cases and healthy controls: one in a case subject and the other in a control subject (both overlapping exons).^30^ The *ROBO1* gene encodes an integral membrane protein that functions in axon guidance and migration of neuronal precursor cell across the midline in the developing brain in rodents. The function of ROBO1 in humans is less well known but it is believed to be important for normal crossing of auditory pathways^51^ and has been implicated in several disorders, including autism,^52^ dyslexia^51^ and phonological buffer deficits.^53^

### Convergence of results?

At first sight, the five genes highlighted as schizophrenia candidate genes in this study seem not to converge in a common cellular pathway. *DLG2* is the only one among these genes to be directly involved in NMDA receptor signaling and it makes an obvious candidate for involvement in schizophrenia pathophysiology. Although it may seem surprising that not more genes belonging to the NMDA receptor pathway were identified in this study, it should be kept in mind that the stringent filters we used allow only the most differentially expressed in the MK801-treated animals to be taken forward to further analysis. This is because of the limitation imposed on the study from the small number of experimental animals. Subsequently, many genes with subtler—although still true—differences in expression have probably been left behind. For example, in our previous study, we found that expression of NMDA receptor subunits was reduced in the MK801-treated rats when testing these specifically.^31^

Looking closer into studies of the function of the remaining four genes reveals several interesting coincidences. First, both FOXP1 and ROBO1 seem to be involved in the complex processes in the brain relating to language and speech. It has long been a popular hypothesis in schizophrenia that it co-evolved with language and is essentially a disorder caused by loss of asymmetry between the brain's hemispheres, which is vital for the separation of speech and thought, and, indeed, auditory hallucinations are among the most common psychotic symptoms in schizophrenia (see, for example, Crow^54^).

Furthermore, SF3B1 is involved in adult neurogenesis, acts as a transcription factors and is associated with PQBP1, which is linked to neurodegenerative disorders and intellectual disability, an endophenotype involved in schizophrenia. Interestingly, *SF3B1* is one of the 108 genes found in the recent largest case–control study on schizophrenia.^27^

In summary, we found 20 genes differentially expressed in a MK801 rat model of schizophrenia and find supporting evidence for five of these genes in the large PGC schizophrenia case–control sample and/or in CNV data from our own case–control sample. This suggests that at least some of these genes are involved in schizophrenia pathology, although definite conclusions cannot yet be made because of the associative basis of the study. Particularly interesting are *SF3B1*, *DLG2* and *FOXP1* because of the strong association for the *SF3B1* in the PGC meta-analysis and the previous evidence implicating *DLG2* and *FOXP1* in schizophrenia and other neurodevelopmental disorders. Our study will hopefully prompt others to focus on these genes in future studies aiming to disentangle the pathologic processes and genetic vulnerabilities underlying schizophrenia.

## Figures and Tables

**Table 1:** Results of an experimental approach to find the best threshold of magnitude (|ALR|) and probability (*P*) of differentially expressed transcripts between MK801-treated and control animals

*|ALR|*	P	*EXP*	*Boot 1*	*Boot 2*	*Boot 3*	*FDR (%)*
0.585	0.05	74	39	12	14	34.5
0.848	0.05	24	15	5	3	41.7
0.585	0.01	15	11	4	2	50.0
0.848	0.01	3	3	1	0	66.7
0.263	0.005	46	25	12	5	40.2
0.585	0.005	4	3	1	2	50.0
0.848	0.005	1	0	0	0	0.0

Abbreviations: |ALR|, absolute average log2 ratio; Boot 1–3, number of probes surpassing the same criteria in permutation comparisons; EXP, number of probes meeting the |ALR| and *P* criteria for difference in expression between MK801- and control animals; FDR, false discovery rate.

**Table 2:** Differentially expressed sequences and the corresponding rat and human genes

*Sequence*	*ALR*^a^	*s.d.*	*Count*^b^	P	*Rat gene*	*Human gene*
AW528011	−1.08	0.24	15	0.00020	*Camk4*^c^	*CAMK4*
AW524430	−0.91	0.57	12	0.028	*Robo1*^c^	*ROBO1*
BF400868	−0.86	0.53	10	0.027	*Mia3*	*MIA3*
BF394251	−0.82	0.53	11	0.031	*Dlg2*	*DLG2*
BF393807	−0.81	0.45	10	0.019	*Smurf2*	*SMURF2*
AI072641	−0.78	0.52	10	0.037	*Foxp1*^c^	*FOXP1*
AW529817	−0.76	0.41	11	0.017	*Palm2*	*PALM2*
NM_022683	−0.74	0.46	9	0.028	*Cul5*	*CUL5*
BI300794	−0.73	0.34	10	0.0085	*Vgll4*^c^	*VGLL4*
BE113314	−0.72	0.37	10	0.013	*Prdm5*	*PRDM5*
NM_133515	−0.71	0.27	12	0.0034	*Akap5*	*AKAP5*
AI234943	−0.69	0.42	10	0.027	*Sf3b1*	*SF3B1*
AI179379	−0.68	0.27	10	0.0041	*Smad2*^c^	*SMAD2*
NM_134326	−0.66	0.30	9	0.0080	*Alb*	*ALB*
AI233106	0.69	0.27	−10	0.0035	*Tslp*^c^	*TSLP*
NM_134392	0.70	0.32	−10	0.0084	*Spata6*	*SPATA6*
NM_053832	0.72	0.34	−11	0.0085	*Foxj1*	*FOXJ1*
NM_024400	0.84	0.38	−11	0.0077	*Adamts1*	*ADAMTS1*
AI070651	0.84	0.40	−12	0.0092	*Wipi1/Arsg*^c^	*WIPI1/ARSG*
BI286012	1.17	0.51	−14	0.0066	*Krt18*	*KRT18*

a Average log2 ratio of expression (MK801 versus control).

b Number of individual MK801- and control animal comparisons with absolute magnitude difference (log2 ratio) of expression >0.585.

c Probe maps immediately downstream of the rat refseq gene sequence in the rat genome (rn6).

**Table 3:** Association of differentially expressed genes with schizophrenia in the PGC sample

*Gene*	*Locus (hg19)*	*No. of markers in PGC_SCZ52*	*Best marker in PGC_SCZ52*	P *(best marker)*	*Locus-wide significance*^a^
*SF3B1*	Chr2:198236697-198319771	318	rs6434928	1.5 × 10^−11^	4.7 × 10^−9^
*FOXP1*	Chr3:70983864-71653140	3431	rs7372960	1.2 × 10^−7^	0.00043
*DLG2*	Chr11:83146055-85358314	13 630	rs12294291	4.9 × 10^−7^	0.0067
*VGLL4*	Chr3:11577540-11782242	1508	chr3_11689216_D	1.4 × 10^−5^	0.021
*ROBO1*	Chr3:78626387-79837059	6944	rs188858898	7.4 × 10^−5^	0.52
*AKAP5*	Chr14:64912216-64961221	253	rs1980520	0.00023	0.057
*PALM2*	Chr9:112383067-112733756	2550	rs78341776	0.00033	0.83
*SMAD2*	Chr18:45339465-45477517	719	rs189158823	0.00045	0.32
*CAMK4*	Chr5:110539946-110840748	1685	rs117821127	0.00099	1
*PRDM5*	Chr4:121595928-121864013	1951	chr4_121723039_I	0.0010	1
*SMURF2*	Chr17:62520734-62678386	644	rs117024269	0.0014	0.92
*CUL5*	Chr11:107859407-107998488	753	chr11_107977411_I	0.0022	1
*ARSG/WIPI1*	Chr17:66235322-66473653	1395	rs149373128	0.0026	1
*SPATA6*	Chr1:48741043-48957880	1306	rs146859685	0.0028	1
*TSLP*	Chr5:110385777-110433722	291	rs79283270	0.011	1
*MIA3*	Chr1:222771443-222861351	448	rs9441835	0.011	1
*ALB*	Chr4:74249971-74307129	278	rs12651581	0.012	1
*ADAMTS1*	Chr21:28188605-28237728	350	rs181607589	0.013	1
*KRT18*	Chr12:53322654-53366685	270	rs113120949	0.014	1
*FOXJ1*	Chr17:74112414-74157380	267	rs117235875	0.014	1

Abbreviation: PGC, Psychiatric Genetics Consortium.

a *P* multiplied by the number of markers at each locus; rs6434928, rs7372960 and rs12294291 remain significantly associated at *P*<0.05 when correcting for all 38 991 markers from the 20 tested loci.
