# Supplementary material for: Expression analysis in a rat psychosis model identifies novel candidate genes validated in a large case–control sample of schizophrenia
Source: Transl Psychiatry. 2015 Oct 13;5(10):e656–. doi: 10.1038/tp.2015.151 (PMC4930128; doi:10.1038/tp.2015.151)
Supplement: Supplementary Information [file tp2015151x1.docx]

**Supplementary information to Expression analysis in a rat psychosis model identifies novel candidate genes validated in a large case-control sample of schizophrenia:**

**Schizophrenia Working Group of the Psychiatric Genomics Consortium**

Stephan Ripke^1,2^, Benjamin M. Neale^1,2,3,4^, Aiden Corvin^5^, James T. R. Walters^6^, Kai-How Farh^1^, Peter A. Holmans^6,7^, Phil Lee^1,2,4^, Brendan Bulik-Sullivan^1,2^, David A. Collier^8,9^, Hailiang Huang^1,3^, Tune H. Pers^3,10,11^, Ingrid Agartz^12,13,14^, Esben Agerbo^15,16,17^, Margot Albus^18^, Madeline Alexander^19^, Farooq Amin^20,21^, Silviu A. Bacanu^22^, Martin Begemann^23^, Richard A Belliveau Jr^2^, Judit Bene^24,25^, Sarah E. Bergen ^2,26^, Elizabeth Bevilacqua^2^, Tim B Bigdeli ^22^, Donald W. Black^27^, Richard Bruggeman^28^, Nancy G. Buccola^29^, Randy L. Buckner^30,31,32^, William Byerley^33^, Wiepke Cahn^34^, Guiqing Cai^35,36^, Murray J. Cairns^39,120,170^, Dominique Campion^37^, Rita M. Cantor^38^, Vaughan J. Carr^39,40^, Noa Carrera^6^, Stanley V. Catts^39,41^, Kimberly D. Chambert^2^, Raymond C. K. Chan^42^, Ronald Y. L. Chen^43^, Eric Y. H. Chen^43,44^, Wei Cheng^45^, Eric F. C. Cheung^46^, Siow Ann Chong^47^, C. Robert Cloninger^48^, David Cohen^49^, Nadine Cohen^50^, Paul Cormican^5^, Nick Craddock^6,7^, Benedicto Crespo-Facorro^210^, James J. Crowley^51^, David Curtis^52,53^, Michael Davidson^54^, Kenneth L. Davis^36^, Franziska Degenhardt^55,56^, Jurgen Del Favero^57^, Lynn E. DeLisi^128,129^ , Ditte Demontis^17,58,59^, Dimitris Dikeos^60^, Timothy Dinan^61^, Srdjan Djurovic^14,62^, Gary Donohoe^5,63^, Elodie Drapeau^36^, Jubao Duan^64,65^, Frank Dudbridge^66^, Naser Durmishi^67^, Peter Eichhammer^68^, Johan Eriksson^69,70,71^, Valentina Escott-Price^6^, Laurent Essioux^72^, Ayman H. Fanous^73,74,75,76^, Martilias S. Farrell^51^, Josef Frank^77^, Lude Franke^78^, Robert Freedman^79^, Nelson B. Freimer^80^, Marion Friedl^81^, Joseph I. Friedman^36^, Menachem Fromer^1,2,4,82^, Giulio Genovese^2^, Lyudmila Georgieva^6^, Elliot S. Gershon^209^, Ina Giegling^81,83^, Paola Giusti-Rodríguez^51^, Stephanie Godard^84^, Jacqueline I. Goldstein^1,3^, Vera Golimbet^85^, Srihari Gopal^86^, Jacob Gratten^87^, Lieuwe de Haan^88^, Christian Hammer^23^, Marian L. Hamshere^6^, Mark Hansen^89^, Thomas Hansen^17,90^, Vahram Haroutunian^36,91,92^, Annette M. Hartmann^81^, Frans A. Henskens^39,93,94^, Stefan Herms^55,56,95^, Joel N. Hirschhorn^3,11,96^, Per Hoffmann^55,56,95^, Andrea Hofman^55,56^, Mads V. Hollegaard^97^, David M. Hougaard^97^, Masashi Ikeda^98^, Inge Joa^99^, Antonio Julià^100^, René S. Kahn^34^, Luba Kalaydjieva^101,102^, Sena Karachanak-Yankova^103^, Juha Karjalainen^78^, David Kavanagh^6^, Matthew C. Keller^104^, Brian J. Kelly^120^, James L. Kennedy^105,106,107^, Andrey Khrunin^108^, Yunjung Kim^51^, Janis Klovins^109^, James A. Knowles^110^, Bettina Konte^81^, Vaidutis Kucinskas^111^, Zita Ausrele Kucinskiene^111^, Hana Kuzelova-Ptackova^112^, Anna K. Kähler^26^, Claudine Laurent^19,113^, Jimmy Lee Chee Keong^47,114^, S. Hong Lee^87^, Sophie E. Legge^6^, Bernard Lerer^115^, Miaoxin Li^43,44,116^ Tao Li^117^, Kung-Yee Liang^118^, Jeffrey Lieberman^119^, Svetlana Limborska^108^, Carmel M. Loughland^39,120^, Jan Lubinski^121^, Jouko Lönnqvist^122^, Milan Macek Jr^112^, Patrik K. E. Magnusson^26^, Brion S. Maher^123^, Wolfgang Maier^124^, Jacques Mallet^125^, Sara Marsal^100^, Manuel Mattheisen^17,58,59,126^, Morten Mattingsdal^14,127^, Robert W. McCarley^128,129^, Colm McDonald^130^, Andrew M. McIntosh^131,132^, Sandra Meier^77^, Carin J. Meijer^88^, Bela Melegh^24,25^, Ingrid Melle^14,133^, Raquelle I. Mesholam-Gately^128,134^, Andres Metspalu^135^, Patricia T. Michie^39,136^, Lili Milani^135^, Vihra Milanova^137^, Younes Mokrab^8^, Derek W. Morris^5,63^, Ole Mors^17,58,138^, Kieran C. Murphy^139^, Robin M. Murray^140^, Inez Myin-Germeys^141^, Bertram Müller-Myhsok^142,143,144^, Mari Nelis^135^, Igor Nenadic^145^, Deborah A. Nertney^146^, Gerald Nestadt^147^, Kristin K. Nicodemus^148^, Liene Nikitina-Zake^109^, Laura Nisenbaum^149^, Annelie Nordin^150^, Eadbhard O’Callaghan^151^, Colm O’Dushlaine^2^, F. Anthony O’Neill^152^, Sang-Yun Oh^153^, Ann Olincy^79^, Line Olsen^17,90^, Jim Van Os^141,154^, Psychosis Endophenotypes International Consortium^155^, Christos Pantelis^39,156^, George N. Papadimitriou^60^, Sergi Papiol^23^, Elena Parkhomenko^36^, Michele T. Pato^110^, Tiina Paunio^157,158^, Milica Pejovic-Milovancevic^159^, Diana O. Perkins^160^, Olli Pietiläinen^158,161^, Jonathan Pimm^53^, Andrew J. Pocklington^6^, John Powell^140^, Alkes Price^3^,^162^, Ann E. Pulver^147^, Shaun M. Purcell^82^, Digby Quested^163^, Henrik B. Rasmussen^17,90^, Abraham Reichenberg^36^, Mark A. Reimers^164^, Alexander L. Richards^6^, Joshua L. Roffman^30,32^, Panos Roussos^82,165^, Douglas M. Ruderfer^6,82^, Veikko Salomaa^71^, Alan R. Sanders^64,65^, Ulrich Schall^39,120^, Christian R. Schubert^166^, Thomas G. Schulze^77,167^, Sibylle G. Schwab^168^, Edward M. Scolnick^2^, Rodney J. Scott^39,169,170^, Larry J. Seidman^128,134^, Jianxin Shi^171^, Engilbert Sigurdsson^172^, Teimuraz Silagadze^173^, Jeremy M. Silverman^36,174^, Kang Sim^47^, Petr Slominsky^108^, Jordan W. Smoller^2,4^, Hon-Cheong So^43^, Chris C. A. Spencer^175^, Eli A. Stahl^3,82^, Hreinn Stefansson^176^, Stacy Steinberg^176^, Elisabeth Stogmann^177^, Richard E. Straub^178^, Eric Strengman^179,34^, Jana Strohmaier^77^, T. Scott Stroup^119^, Mythily Subramaniam^47^, Jaana Suvisaari^122^, Dragan M. Svrakic^48^, Jin P. Szatkiewicz^51^, Erik Söderman^12^, Srinivas Thirumalai^180^, Draga Toncheva^103^, Paul A. Tooney^39,120,170^ , Sarah Tosato^181^, Juha Veijola^182,183^, John Waddington^184^, Dermot Walsh^185^, Dai Wang^86^, Qiang Wang^117^, Bradley T. Webb^22^, Mark Weiser^54^, Dieter B. Wildenauer^186^, Nigel M. Williams^6^, Stephanie Williams^51^, Stephanie H. Witt^77^, Aaron R. Wolen^164^, Emily H. M. Wong^43^, Brandon K. Wormley^22^, Jing Qin Wu^39,170^, Hualin Simon Xi^187^, Clement C. Zai^105,106^, Xuebin Zheng^188^, Fritz Zimprich^177^, Naomi R. Wray^87^, Kari Stefansson^176^, Peter M. Visscher^87^, Wellcome Trust Case-Control Consortium 2^189^, Rolf Adolfsson^150^, Ole A. Andreassen^14,133^, Douglas H. R. Blackwood^132^, Elvira Bramon^190^, Joseph D. Buxbaum^35,36,91,191^, Anders D. Børglum^17,58,59,138^, Sven Cichon^55,56,95,192^, Ariel Darvasi^193^, Enrico Domenici^194^, Hannelore Ehrenreich^23^, Tõnu Esko^3,11,96,135^, Pablo V. Gejman^64,65^, Michael Gill^5^, Hugh Gurling^53^, Christina M. Hultman^26^, Nakao Iwata^98^, Assen V. Jablensky^39,102,186,195^, Erik G. Jönsson^12,14^, Kenneth S. Kendler^196^, George Kirov^6^, Jo Knight^105,106,107^, Todd Lencz^197,198,199^, Douglas F. Levinson^19^, Qingqin S. Li^86^, Jianjun Liu^188,200^, Anil K. Malhotra^197,198,199^, Steven A. McCarroll^2,96^, Andrew McQuillin^53^, Jennifer L. Moran^2^, Preben B. Mortensen^15,16,17^, Bryan J. Mowry^87,201^, Markus M. Nöthen^55,56^, Roel A. Ophoff^38,80,34^, Michael J. Owen^6,7^, Aarno Palotie^2,4,161^, Carlos N. Pato^110^, Tracey L. Petryshen^2,128,202^, Danielle Posthuma^203,204,205^, Marcella Rietschel^77^, Brien P. Riley^196^, Dan Rujescu^81,83^, Pak C. Sham^43,44,116^ Pamela Sklar^82,91,165^, David St Clair^206^, Daniel R. Weinberger^178,207^, Jens R. Wendland^166^, Thomas Werge^17,90,208^, Mark J. Daly^1,2,3^, Patrick F. Sullivan^26,51,160^ & Michael C. O’Donovan^6,7^

^1^Analytic and Translational Genetics Unit, Massachusetts General Hospital, Boston, Massachusetts 02114, USA.

^2^Stanley Center for Psychiatric Research, Broad Institute of MIT and Harvard, Cambridge, Massachusetts 02142, USA.

^3^Medical and Population Genetics Program, Broad Institute of MIT and Harvard, Cambridge, Massachusetts 02142, USA.

^4^Psychiatric and Neurodevelopmental Genetics Unit, Massachusetts General Hospital, Boston, Massachusetts 02114, USA.

^5^Neuropsychiatric Genetics Research Group, Department of Psychiatry, Trinity College Dublin, Dublin 8, Ireland.

^6^MRC Centre for Neuropsychiatric Genetics and Genomics, Institute of Psychological Medicine and Clinical Neurosciences, School of Medicine, Cardiff University, Cardiff, CF24 4HQ, UK.

^7^National Centre for Mental Health, Cardiff University, Cardiff, CF24 4HQ, UK.

^8^Eli Lilly and Company Limited, Erl Wood Manor, Sunninghill Road, Windlesham, Surrey, GU20 6PH, UK. ^9^Social, Genetic and Developmental Psychiatry Centre, Institute of Psychiatry, King’s College London, London, SE5 8AF, UK.

^10^Center for Biological Sequence Analysis, Department of Systems Biology, Technical University of Denmark, DK-2800, Denmark.

^11^Division of Endocrinology and Center for Basic and Translational Obesity Research, Boston Children’s Hospital, Boston, Massachusetts, 02115USA.

^12^Department of Clinical Neuroscience, Psychiatry Section, Karolinska Institutet, SE-17176 Stockholm, Sweden. ^13^Department of Psychiatry, Diakonhjemmet Hospital, 0319 Oslo, Norway.

^14^NORMENT, KG Jebsen Centre for Psychosis Research, Institute of Clinical Medicine, University of Oslo, 0424 Oslo, Norway.

^15^Centre for Integrative Register-based Research, CIRRAU, Aarhus University, DK-8210 Aarhus, Denmark.

^16^National Centre for Register-based Research, Aarhus University, DK-8210 Aarhus, Denmark.

^17^The Lundbeck Foundation Initiative for Integrative Psychiatric Research, iPSYCH, Denmark.

^18^State Mental Hospital, 85540 Haar, Germany.

^19^Department of Psychiatry and Behavioral Sciences, Stanford University, Stanford, California 94305, USA.

^20^Department of Psychiatry and Behavioral Sciences, Atlanta Veterans Affairs Medical Center, Atlanta, Georgia 30033, USA.

^21^Department of Psychiatry and Behavioral Sciences, Emory University, Atlanta Georgia 30322, USA.

^22^Virginia Institute for Psychiatric and Behavioral Genetics, Department of Psychiatry, Virginia Commonwealth University, Richmond, Virginia 23298, USA.

^23^Clinical Neuroscience, Max Planck Institute of Experimental Medicine, Göttingen 37075, Germany.

^24^Department of Medical Genetics, University of Pécs, Pécs H-7624, Hungary.

^25^Szentagothai Research Center, University of Pécs, Pécs H-7624, Hungary.

^26^Department of Medical Epidemiology and Biostatistics, Karolinska Institutet, Stockholm SE-17177, Sweden.

^27^Department of Psychiatry, University of Iowa Carver College of Medicine, Iowa City, Iowa 52242, USA.

^28^University Medical Center Groningen, Department of Psychiatry, University of Groningen NL-9700 RB, The Netherlands.

^29^School of Nursing, Louisiana State University Health Sciences Center, New Orleans, Louisiana 70112, USA.

^30^Athinoula A. Martinos Center, Massachusetts General Hospital, Boston, Massachusetts 02129, USA.

^31^Center for Brain Science, Harvard University, Cambridge, Massachusetts, 02138 USA.

^32^Department of Psychiatry, Massachusetts General Hospital, Boston, Massachusetts, 02114 USA.

^33^Department of Psychiatry, University of California at San Francisco, San Francisco, California, 94143 USA.

^34^University Medical Center Utrecht, Department of Psychiatry, Rudolf Magnus Institute of Neuroscience, 3584 Utrecht, The Netherlands.

^35^Department of Human Genetics, Icahn School of Medicine at Mount Sinai, New York, New York 10029 USA.

^36^Department of Psychiatry, Icahn School of Medicine at Mount Sinai, New York, New York 10029 USA.

^37^Centre Hospitalier du Rouvray and INSERM U1079 Faculty of Medicine, 76301 Rouen, France.

^38^Department of Human Genetics, David Geffen School of Medicine, University of California, Los Angeles, California 90095, USA.

^39^Schizophrenia Research Institute, Sydney NSW 2010, Australia.

^40^School of Psychiatry, University of New South Wales, Sydney NSW 2031, Australia.

^41^Royal Brisbane and Women’s Hospital, University of Queensland, Brisbane, St Lucia QLD 4072, Australia.

^42^Institute of Psychology, Chinese Academy of Science, Beijing 100101, China.

^43^Department of Psychiatry, Li Ka Shing Faculty of Medicine, The University of Hong Kong, Hong Kong, China.

^44^State Key Laboratory for Brain and Cognitive Sciences, Li Ka Shing Faculty of Medicine, The University of Hong Kong, Hong Kong, China.

^45^Department of Computer Science, University of North Carolina, Chapel Hill, North Carolina 27514, USA.

^46^Castle Peak Hospital, Hong Kong, China.

^47^Institute of Mental Health, Singapore 539747, Singapore.

^48^Department of Psychiatry, Washington University, St. Louis, Missouri 63110, USA.

^49^Department of Child and Adolescent Psychiatry, Assistance Publique Hopitaux de Paris, Pierre and Marie Curie Faculty of Medicine and Institute for Intelligent Systems and Robotics, Paris, 75013, France.

^50^ Blue Note Biosciences, Princeton, New Jersey 08540, USA

^51^Department of Genetics, University of North Carolina, Chapel Hill, North Carolina 27599-7264, USA.

^52^Department of Psychological Medicine, Queen Mary University of London, London E1 1BB, UK.

^53^Molecular Psychiatry Laboratory, Division of Psychiatry, University College London, London WC1E 6JJ, UK.

^54^Sheba Medical Center, Tel Hashomer 52621, Israel.

^55^Department of Genomics, Life and Brain Center, D-53127 Bonn, Germany.

^56^Institute of Human Genetics, University of Bonn, D-53127 Bonn, Germany.

^57^Applied Molecular Genomics Unit, VIB Department of Molecular Genetics, University of Antwerp, B-2610 Antwerp, Belgium.

^58^Centre for Integrative Sequencing, iSEQ, Aarhus University, DK-8000 Aarhus C, Denmark.

^59^Department of Biomedicine, Aarhus University, DK-8000 Aarhus C, Denmark.

^60^First Department of Psychiatry, University of Athens Medical School, Athens 11528, Greece.

^61^Department of Psychiatry, University College Cork, Co. Cork, Ireland.

^62^Department of Medical Genetics, Oslo University Hospital, 0424 Oslo, Norway.

^63^Cognitive Genetics and Therapy Group, School of Psychology and Discipline of Biochemistry, National University of Ireland Galway, Co. Galway, Ireland.

^64^Department of Psychiatry and Behavioral Neuroscience, University of Chicago, Chicago, Illinois 60637, USA.

^65^Department of Psychiatry and Behavioral Sciences, NorthShore University HealthSystem, Evanston, Illinois 60201, USA.

^66^Department of Non-Communicable Disease Epidemiology, London School of Hygiene and Tropical Medicine, London WC1E 7HT, UK.

^67^Department of Child and Adolescent Psychiatry, University Clinic of Psychiatry, Skopje 1000, Republic of Macedonia.

^68^Department of Psychiatry, University of Regensburg, 93053 Regensburg, Germany.

^69^Department of General Practice, Helsinki University Central Hospital, University of Helsinki P.O. Box 20, Tukholmankatu 8 B, FI-00014, Helsinki, Finland

^70^Folkhälsan Research Center, Helsinki, Finland, Biomedicum Helsinki 1, Haartmaninkatu 8, FI-00290, Helsinki, Finland.

^71^National Institute for Health and Welfare, P.O. BOX 30, FI-00271 Helsinki, Finland.

^72^Translational Technologies and Bioinformatics, Pharma Research and Early Development, F. Hoffman-La Roche, CH-4070 Basel, Switzerland.

^73^Department of Psychiatry, Georgetown University School of Medicine, Washington DC 20057, USA.

^74^Department of Psychiatry, Keck School of Medicine of the University of Southern California, Los Angeles, California 90033, USA.

^75^Department of Psychiatry, Virginia Commonwealth University School of Medicine, Richmond, Virginia 23298, USA.

^76^Mental Health Service Line, Washington VA Medical Center, Washington DC 20422, USA.

^77^Department of Genetic Epidemiology in Psychiatry, Central Institute of Mental Health, Medical Faculty Mannheim, University of Heidelberg, Heidelberg , D-68159 Mannheim, Germany.

^78^Department of Genetics, University of Groningen, University Medical Centre Groningen, 9700 RB Groningen, The Netherlands.

^79^Department of Psychiatry, University of Colorado Denver, Aurora, Colorado 80045, USA.

^80^Center for Neurobehavioral Genetics, Semel Institute for Neuroscience and Human Behavior, University of California, Los Angeles, California 90095, USA.

^81^Department of Psychiatry, University of Halle, 06112 Halle, Germany.

^82^Division of Psychiatric Genomics, Department of Psychiatry, Icahn School of Medicine at Mount Sinai, New York, New York 10029, USA.

^83^Department of Psychiatry, University of Munich, 80336, Munich, Germany.

^84^Departments of Psychiatry and Human and Molecular Genetics, INSERM, Institut de Myologie, Hôpital de la Pitiè-Salpêtrière, Paris, 75013, France.

^85^Mental Health Research Centre, Russian Academy of Medical Sciences, 115522 Moscow, Russia.

^86^Neuroscience Therapeutic Area, Janssen Research and Development, Raritan, New Jersey 08869, USA.

^87^Queensland Brain Institute, The University of Queensland, Brisbane, Queensland, QLD 4072, Australia.

^88^Academic Medical Centre University of Amsterdam, Department of Psychiatry, 1105 AZ Amsterdam, The Netherlands.

^89^Illumina, La Jolla, California, California 92122, USA.

^90^Institute of Biological Psychiatry, Mental Health Centre Sct. Hans, Mental Health Services Copenhagen, DK-4000, Denmark.

^91^Friedman Brain Institute, Icahn School of Medicine at Mount Sinai, New York, New York 10029, USA.

^92^J. J. Peters VA Medical Center, Bronx, New York, New York 10468, USA.

^93^Priority Research Centre for Health Behaviour, University of Newcastle, Newcastle NSW 2308, Australia.

^94^School of Electrical Engineering and Computer Science, University of Newcastle, Newcastle NSW 2308, Australia.

^95^Division of Medical Genetics, Department of Biomedicine, University of Basel, Basel, CH-4058, Switzerland.

^96^Department of Genetics, Harvard Medical School, Boston, Massachusetts 02115, USA.

^97^Section of Neonatal Screening and Hormones, Department of Clinical Biochemistry, Immunology and Genetics, Statens Serum Institut, Copenhagen, DK-2300, Denmark.

^98^Department of Psychiatry, Fujita Health University School of Medicine, Toyoake, Aichi, 470-1192, Japan.

^99^Regional Centre for Clinical Research in Psychosis, Department of Psychiatry, Stavanger University Hospital, 4011 Stavanger, Norway.

^100^Rheumatology Research Group, Vall d'Hebron Research Institute, Barcelona, 08035, Spain.

^101^Centre for Medical Research, The University of Western Australia, Perth, WA 6009, Australia.

^102^The Perkins Institute for Medical Research, The University of Western Australia, Perth, WA 6009, Australia.

^103^Department of Medical Genetics, Medical University, Sofia1431, Bulgaria.

^104^Department of Psychology, University of Colorado Boulder, Boulder, Colorado 80309, USA.

^105^Campbell Family Mental Health Research Institute, Centre for Addiction and Mental Health, Toronto, Ontario, M5T 1R8, Canada.

^106^Department of Psychiatry, University of Toronto, Toronto, Ontario, M5T 1R8, Canada.

^107^Institute of Medical Science, University of Toronto, Toronto, Ontario, M5S 1A8, Canada.

^108^Institute of Molecular Genetics, Russian Academy of Sciences, Moscow123182, Russia.

^109^Latvian Biomedical Research and Study Centre, Riga, LV-1067, Latvia.

^110^Department of Psychiatry and Zilkha Neurogenetics Institute, Keck School of Medicine at University of Southern California, Los Angeles, California 90089, USA.

^111^Faculty of Medicine, Vilnius University, LT-01513 Vilnius, Lithuania.

^112^ Department of Biology and Medical Genetics, 2nd Faculty of Medicine and University Hospital Motol, 150 06 Prague, Czech Republic.

^113^ Department of Child and Adolescent Psychiatry, Pierre and Marie Curie Faculty of Medicine, Paris 75013, France.

^114^Duke-NUS Graduate Medical School, Singapore 169857, Singapore.

^115^Department of Psychiatry, Hadassah-Hebrew University Medical Center, Jerusalem 91120, Israel.

^116^Centre for Genomic Sciences, The University of Hong Kong, Hong Kong, China.

^117^Mental Health Centre and Psychiatric Laboratory, West China Hospital, Sichuan University, Chengdu, 610041, Sichuan, China.

^118^Department of Biostatistics, Johns Hopkins University Bloomberg School of Public Health, Baltimore, Maryland 21205, USA.

^119^Department of Psychiatry, Columbia University, New York, New York 10032, USA.

^120^Priority Centre for Translational Neuroscience and Mental Health, University of Newcastle, Newcastle NSW 2300, Australia.

^121^Department of Genetics and Pathology, International Hereditary Cancer Center, Pomeranian Medical University in Szczecin, 70-453 Szczecin, Poland.

^122^Department of Mental Health and Substance Abuse Services; National Institute for Health and Welfare, P.O. BOX 30, FI-00271 Helsinki, Finland

^123^Department of Mental Health, Bloomberg School of Public Health, Johns Hopkins University, Baltimore, Maryland 21205, USA.

^124^Department of Psychiatry, University of Bonn, D-53127 Bonn, Germany.

^125^Centre National de la Recherche Scientifique, Laboratoire de Génétique Moléculaire de la Neurotransmission et des Processus Neurodégénératifs, Hôpital de la Pitié Salpêtrière, 75013, Paris, France.

^126^Department of Genomics Mathematics, University of Bonn, D-53127 Bonn, Germany.

^127^Research Unit, Sørlandet Hospital, 4604 Kristiansand, Norway.

^128^Department of Psychiatry, Harvard Medical School, Boston, Massachusetts 02115, USA.

^129^VA Boston Health Care System, Brockton, Massachusetts 02301, USA.

^130^Department of Psychiatry, National University of Ireland Galway, Co. Galway, Ireland.

^131^Centre for Cognitive Ageing and Cognitive Epidemiology, University of Edinburgh, Edinburgh EH16 4SB, UK.

^132^Division of Psychiatry, University of Edinburgh, Edinburgh EH16 4SB, UK.

^133^Division of Mental Health and Addiction, Oslo University Hospital, 0424 Oslo, Norway.

^134^Massachusetts Mental Health Center Public Psychiatry Division of the Beth Israel Deaconess Medical Center, Boston, Massachusetts 02114, USA.

^135^Estonian Genome Center, University of Tartu, Tartu 50090, Estonia.

^136^School of Psychology, University of Newcastle, Newcastle NSW 2308, Australia.

^137^First Psychiatric Clinic, Medical University, Sofia 1431, Bulgaria.

^138^Department P, Aarhus University Hospital, DK-8240 Risskov, Denmark.

^139^Department of Psychiatry, Royal College of Surgeons in Ireland, Dublin 2, Ireland.

^140^King’s College London, London SE5 8AF, UK.

^141^Maastricht University Medical Centre, South Limburg Mental Health Research and Teaching Network, EURON, 6229 HX Maastricht, The Netherlands.

^142^Institute of Translational Medicine, University of Liverpool, Liverpool L69 3BX, UK.

^143^Max Planck Institute of Psychiatry, 80336 Munich, Germany.

^144^Munich Cluster for Systems Neurology (SyNergy), 80336 Munich, Germany.

^145^Department of Psychiatry and Psychotherapy, Jena University Hospital, 07743 Jena, Germany.

^146^Department of Psychiatry, Queensland Brain Institute and Queensland Centre for Mental Health Research, University of Queensland, Brisbane, Queensland, St Lucia QLD 4072, Australia.

^147^Department of Psychiatry and Behavioral Sciences, Johns Hopkins University School of Medicine, Baltimore, Maryland 21205, USA.

^148^Department of Psychiatry, Trinity College Dublin, Dublin 2, Ireland.

^149^Eli Lilly and Company, Lilly Corporate Center, Indianapolis, 46285 Indiana, USA.

^150^Department of Clinical Sciences, Psychiatry, Umeå University, SE-901 87 Umeå, Sweden.

^151^DETECT Early Intervention Service for Psychosis, Blackrock, Co. Dublin, Ireland.

^152^Centre for Public Health, Institute of Clinical Sciences, Queen’s University Belfast, Belfast BT12 6AB, UK.

^153^Lawrence Berkeley National Laboratory, University of California at Berkeley, Berkeley, California 94720, USA.

^154^Institute of Psychiatry, King’s College London, London SE5 8AF, UK.

^155^A list of authors and affiliations appear in the Supplementary Information.

^156^Melbourne Neuropsychiatry Centre, University of Melbourne & Melbourne Health, Melbourne, Vic 3053, Australia.

^157^Department of Psychiatry, University of Helsinki, P.O. Box 590, FI-00029 HUS, Helsinki, Finland.

^158^Public Health Genomics Unit, National Institute for Health and Welfare, P.O. BOX 30, FI-00271 Helsinki, Finland.

^159^Medical Faculty, University of Belgrade, 11000 Belgrade, Serbia.

^160^Department of Psychiatry, University of North Carolina, Chapel Hill, North Carolina 27599-7160, USA.

^161^Institute for Molecular Medicine Finland, FIMM, University of Helsinki, P.O. Box 20

FI-00014, Helsinki, Finland.

^162^Department of Epidemiology, Harvard School of Public Health, Boston, Massachusetts 02115, USA.

^163^Department of Psychiatry, University of Oxford, Oxford, OX3 7JX, UK.

^164^Virginia Institute for Psychiatric and Behavioral Genetics, Virginia Commonwealth University, Richmond, Virginia 23298, USA.

^165^Institute for Multiscale Biology, Icahn School of Medicine at Mount Sinai, New York, New York 10029, USA.

^166^PharmaTherapeutics Clinical Research, Pfizer Worldwide Research and Development, Cambridge, Massachusetts 02139, USA.

^167^Department of Psychiatry and Psychotherapy, University of Gottingen, 37073 Göttingen, Germany.

^168^Psychiatry and Psychotherapy Clinic, University of Erlangen, 91054 Erlangen, Germany.

^169^Hunter New England Health Service, Newcastle NSW 2308, Australia.

^170^School of Biomedical Sciences and Pharmacy, University of Newcastle, Callaghan NSW 2308, Australia.

^171^Division of Cancer Epidemiology and Genetics, National Cancer Institute, Bethesda, Maryland 20892, USA.

^172^University of Iceland, Landspitali, National University Hospital, 101 Reykjavik, Iceland.

^173^Department of Psychiatry and Drug Addiction, Tbilisi State Medical University (TSMU), **N33, 0177** Tbilisi, Georgia.

^174^Research and Development, Bronx Veterans Affairs Medical Center, New York, New York 10468, USA.

^175^Wellcome Trust Centre for Human Genetics, Oxford, OX3 7BN, UK.

^176^deCODE Genetics, 101 Reykjavik, Iceland.

^177^Department of Clinical Neurology, Medical University of Vienna, 1090 Wien, Austria.

^178^Lieber Institute for Brain Development, Baltimore, Maryland 21205, USA.

^179^Department of Medical Genetics, University Medical Centre Utrecht, Universiteitsweg 100, 3584 CG, Utrecht, The Netherlands.

^180^Berkshire Healthcare NHS Foundation Trust, Bracknell RG12 1BQ, UK.

^181^Section of Psychiatry, University of Verona, 37134 Verona, Italy.

^182^Department of Psychiatry, University of Oulu, P.O. BOX 5000, 90014, Finland

^183^University Hospital of Oulu, P.O.BOX 20, 90029 OYS, Finland.

^184^Molecular and Cellular Therapeutics, Royal College of Surgeons in Ireland, Dublin 2, Ireland.

^185^Health Research Board, Dublin 2, Ireland.

^186^School of Psychiatry and Clinical Neurosciences, The University of Western Australia, Perth WA6009, Australia.

^187^Computational Sciences CoE, Pfizer Worldwide Research and Development, Cambridge, Massachusetts 02139, USA.

^188^Human Genetics, Genome Institute of Singapore, A*STAR, Singapore 138672, Singapore.

^189^A list of authors and affiliations appear in the Supplementary Information.

^190^University College London, London WC1E 6BT, UK.

^191^Department of Neuroscience, Icahn School of Medicine at Mount Sinai, New York, New York 10029, USA.

^192^Institute of Neuroscience and Medicine (INM-1), Research Center Juelich, 52428 Juelich, Germany.

^193^Department of Genetics, The Hebrew University of Jerusalem, 91905 Jerusalem, Israel.

^194^Neuroscience Discovery and Translational Area, Pharma Research and Early Development, F. Hoffman-La Roche, CH-4070 Basel, Switzerland.

^195^Centre for Clinical Research in Neuropsychiatry, School of Psychiatry and Clinical Neurosciences, The University of Western Australia, Medical Research Foundation Building, Perth WA 6000, Australia.

^196^Virginia Institute for Psychiatric and Behavioral Genetics, Departments of Psychiatry and Human and Molecular Genetics, Virginia Commonwealth University, Richmond, Virginia 23298, USA.

^197^The Feinstein Institute for Medical Research, Manhasset, New York, 11030 USA.

^198^The Hofstra NS-LIJ School of Medicine, Hempstead, New York, 11549 USA.

^199^The Zucker Hillside Hospital, Glen Oaks, New York,11004 USA.

^200^Saw Swee Hock School of Public Health, National University of Singapore, Singapore 117597, Singapore.

^201^Queensland Centre for Mental Health Research, University of Queensland, Brisbane 4076, Queensland, Australia.

^202^Center for Human Genetic Research and Department of Psychiatry, Massachusetts General Hospital, Boston, Massachusetts 02114, USA.

^203^Department of Child and Adolescent Psychiatry, Erasmus University Medical Centre, Rotterdam 3000, The Netherlands.

^204^Department of Complex Trait Genetics, Neuroscience Campus Amsterdam, VU University Medical Center Amsterdam, Amsterdam 1081, The Netherlands.

^205^Department of Functional Genomics, Center for Neurogenomics and Cognitive Research, Neuroscience Campus Amsterdam, VU University, Amsterdam 1081, The Netherlands.

^206^University of Aberdeen, Institute of Medical Sciences, Aberdeen, AB25 2ZD, UK.

^207^Departments of Psychiatry, Neurology, Neuroscience and Institute of Genetic Medicine, Johns Hopkins School of Medicine, Baltimore, Maryland 21205, USA.

^208^Department of Clinical Medicine, University of Copenhagen, Copenhagen 2200, Denmark.

^209^Departments of Psychiatry and Human Genetics, University of Chicago, Chicago, Illinois 60637, USA.

^210^University Hospital Marqués de Valdecilla, Instituto de Formación e Investigación Marqués de Valdecilla, University of Cantabria, E‐39008 Santander, Spain.
